# Immunological and morphological analysis of heterotopic ossification differs to healthy controls

**DOI:** 10.1186/s12891-018-2246-9

**Published:** 2018-09-11

**Authors:** Klemens Trieb, Andreas Meryk, Sascha Senck, Erin Naismith, Beatrix Grubeck-Loebenstein

**Affiliations:** 1 Department of Orthopaedics, Klinikum Wels-Grieskirchen, Grieskirchnerstr 42, 4600 Wels, Austria; 2 Institute for Biomedical Aging Research, University of Innsbruck, 5020 Innsbruck, Austria; 3 Computed Tomography Research Group, University of Applied Sciences Upper Austria, 4600 Wels, Austria

## Abstract

**Background:**

Formation of lamellar bone in non-osseus tissue is a pathological process called heterotopic ossification. It is the aim of this study to analyse the morphology and immunological status of patients with heterotopic ossification compared to individual healthy persons.

**Methods:**

Human bone marrow and blood samples were obtained from 6 systemically healthy individuals and 4 patients during resection of heterotopic ossification from bone at hip arthroplasty. Bone was fragmented and treated with purified collagenase. Immunofluorescence surface staining was performed and analyzed with flow cytometry. Microcomputed tomography scanning was done performed at a resolution of 11 and 35 μm isometric voxel size respectively using a two different cone beam X-computer tomography systems and a microfocus X-ray tube. Subsequently the volume data was morphometrically analysed.

**Results:**

The monocytes, stem cells, stroma cells and granulocytes progenitor cells were strongly reduced in the heterotopic ossification patient. Additionally a significant reduction of stromal stem cells cells and CD34 positive stem cells was observed. The frequency of NK-cells, B cells and T cells were not altered in the patients with heterotopic ossification compared to a healthy person. Micromorphometric parameters showed a lower content of mineralized bone tissue compared to normal bone. Mean trabecular thickness showed a high standard deviation, indicating a high variation in trabecular thickness, anisotropy and reducing bone strength.

**Conclusions:**

This work shows altered immunological distribution that is accompanied by a low decrease in bone volume fraction and tissue mineral density in the heterotopic ossification sample compared to normal bone. Compared to healthy subjects, this might reflect an immunological participation in the development of this entity.

## Background

Formation of lamellar bone in non-osseus tissue is a pathological process called heterotopic ossification (HO). This can occur in muscle or connective tissue as a result of trauma, surgery, fractures, neurological injury or genetic mutations (fibrodysplasia ossificans progressiva, Albright’s hereditary osteodystrophy). It causes major clinical burdens due to limitation of motion, persistent pain and nerve entrapement [1–4]. So far morphometric data on morphometric indices like porosity, tissue mineral density, and trabecular volume is fragmentary for human samples and immunological data are rarely available. Skeletal muscle tissue has a wide capacity for regenaration by myogenic stem cells in combination with mesenchymal stromal cells. It is not clear which factors induce enchondral bone formation during this process. Some studies have proposed endothelial or brown adipogenic cells as a or the source for HO. The reciprocal interactions between bone and the immune system have become more the subject of increased attention in recent years and the so called osteoimmunology describes cytokine induces bone resorption and inflammatory induced ossification [5–14].

Neurogenic HO induced by spinal cord or traumatic brain injury is described but detailed characteristion of immunologigal and morphologic changes are hardly available. It is the aim of this study to analyse the morphology and immunological status of patients with heterotopic ossification compared to individual healthy persons [15].

## Methods

### Patients

#### Human sample collection and preparation

Heterotopic ossification tissue was obtained at resection from four patients and divided for further analysis. The first was obtained from male in the beginning 50s patient who suffered a central ganglion bleeding one year before and developed a central nervous system induced HO in the left musculus vastus. The second was obtained from man in the end 60s suffering from a postdicectomy ischiadical lesion developing peripheral neuroathy induced HO after hip arthroplsaty. The third was obtained from woman in the beginning 20s developing HO after fixation of a femoral neck fracture without neurological impairment. The fourth healthy patient underwent hip replacement (male mid 50s) after an old femur fracture with a removed intramedullary nail. In the gluteus he had a HTO which had to be removed for hip approach, so we gained normal bone (femoral head) and HTO from one patient for analysis. Human bone marrow (BM) samples were obtained from age matched systemically healthy individuals (4 male, 2 female, mean age 52 years) who did not receive immunomodulatory drugs or suffer from diseases known to influence the immune system, including autoimmune diseases and cancer. Informed consent for test and publication was given and documented from each patient after the study received approval of the local institution of the corresponding author and none of the authors has competing interests according to BioMed Central’s guidance. Total hip arthroplasty was performed by an antero-lateral minimal invasive approach and bone was harvested from the resected neck and femoral head to isolate bone marrow mononuclear cells (BMMCs) [16]. Bone fragments were washed once with complete RPMI medium (RPMI 1640 supplemented with 10% FCS, 100 U/ml penicillin, and 100 μg/ml streptomycin; Invitrogen) and treated with purified collagenase (CLSPA, Worthington Biochemical; 20 U/ml in complete RPMI medium) for 1 h at 37 °C. After centrifugation purification of BMMCs was done by density gradient centrifugation (Ficoll-Hypaque). This methods are described in detail in previous studies [17].

#### Flow cytometry

Immunofluorescence surface staining was performed by adding a panel of directly conjugated antibodies to freshly prepared BMMCs. Labelled cells were measured by a FACSCanto II (BD Biosciences) and analyzed with Flowjo.

#### Microcomputed tomography

During the scanning procedure, fresh samples were stored in air-sealed polymer sample holders to prevent dehydration. The complete samples were scanned at a resolution of 35 μm isometric voxel size using a RayScan 250E cone beam XCT device equipped with a Perkin Elmer flat panel detector (2048 × 2048 pixels with a pixel size 200 μm) and a Viscom 225 kV microfocus X-ray tube. The X-ray scanning parameters were set to 120 kV and 420 μA with an integration time of 1500 ms; a 0.5 mm thick copper filter-plate was applied to prevent beam hardening artefacts. Hydroxyapatite rods (HA; 8 mm diameter, 250 and 750 mg HA/cm^3^) were scanned in the same sealed specimen holder to calibrate images for 1) tissue mineral density (TMD) of the trabecular bone to quantifiy trabecula mineralization and 2) bone mineral density (BMD) of trabecular bone in conjunction with the surrounding soft tissue.

A second scan was conducted on cut out samples (ca. 12 mm in diameter) of the respective specimen at resolution of 11 μm isometric voxel size using a GE Phoenix Nanotom 180 cone beam XCT device equipped with a panel detector (2300 × 2300 pixels) and a 180 kV nanofocus X-ray tube. The X-ray scanning parameters were set to 80 kV and 230 μA with an integration time of 600 ms. Image information for each data set was separated into tissue and background using the “advanced threshold” function using Volume Graphics 2.2. Subsequently, volume data was transferred to CTAn (Version 1.16; Bruker) for morphometric analysis. Calculated morphometric indices include bone volume fraction (BV/TV, bone volume/total volume), mean trabecular thickness (TbTh.mean), standard deviation of trabecular thickness (TbTh.SD), mean trabecular separation (TbSp.mean), standard deviation of trabecular separation (TbSp.SD), degree of anisotropy (DA), and connectivity (Con). The computation of these indices is implemented in CTAn and is based on the work of Hildebrand and Ruegsegger [18] and Remy and Thiel [19]. Moreover, TMD and BMD were computed using a calibration curve based on the 16-bit grey values of the two above mentioned Hydroxyapatite rods.

#### Histology

For histology, formalin-fixed HO tissue was decalcified and embedded in methyl metacrylate. Sections (6 μm) were cut, deplastified and stained with Goldner trichrome for comparative histology.

### Statistical analysis

The data obtained in the study are following a non-parametric distribution. Therefore statistical significance was assessed by Spearman correlation analysis, Mann–Whitney test and Wilcoxon matched pairs test, a *p*-value of less than 0.05 was considered as significant. All data are shown as mean ± standard error of the mean (SEM). Statistical analysis was performed using GraphPad Prism software version 5.0 (GraphPad Software). To determine the significance of differences between two groups, the unpaired two-tailed t test were used, as indicated in the figure legends.

## Results

### Morphology and histology

**Fig. 1:**
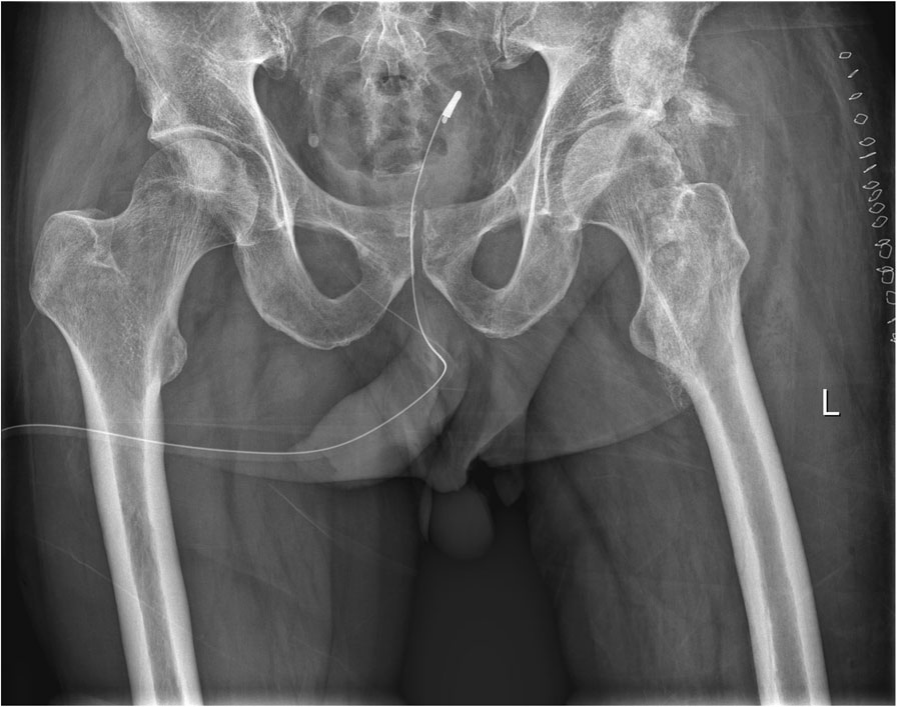
Radiograph of the HTO of the left hip

**Fig. 2:**
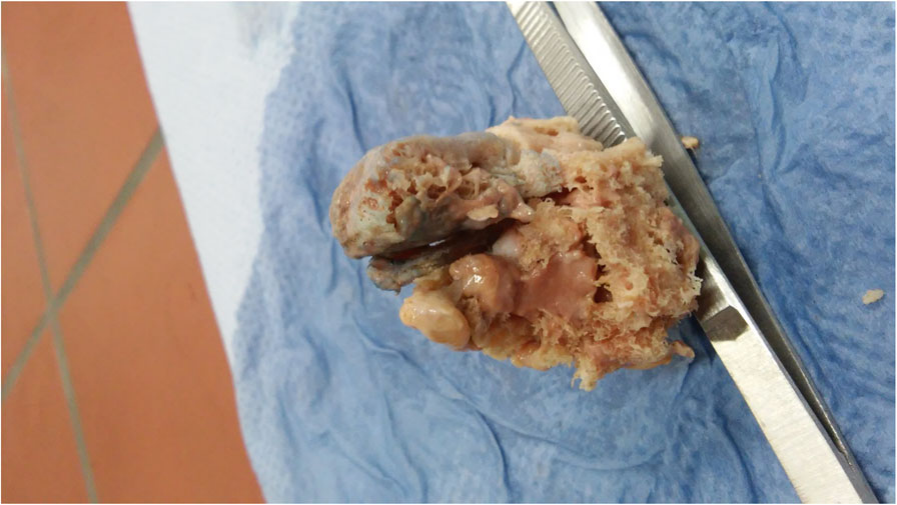
Photograph of the HO after resection and before splitting for different experiments

**Fig. 3:**
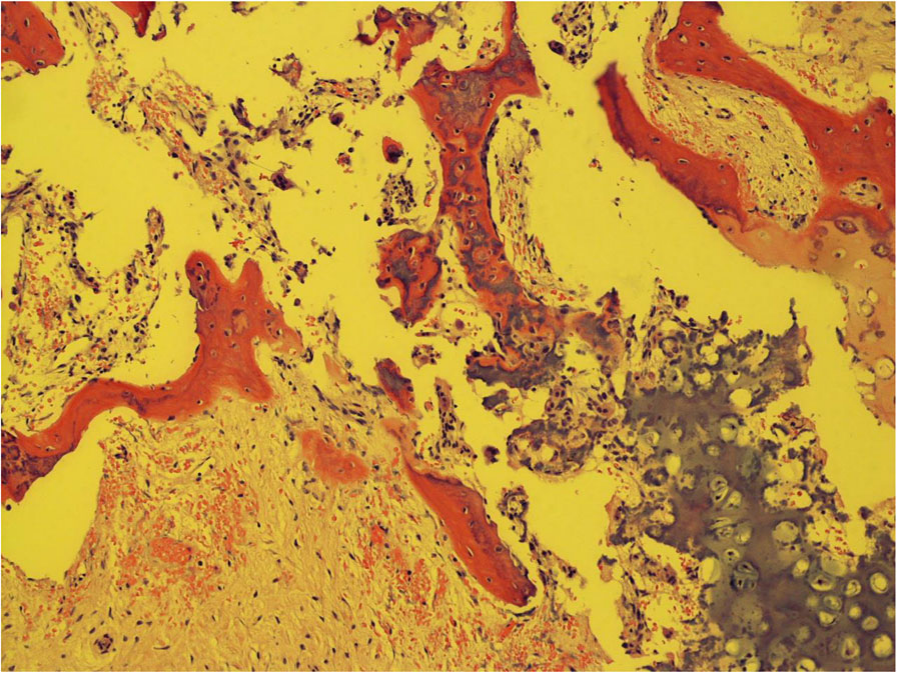
Histological picture (haematoxilin eosin × 100)

The heterotopic ossification is characterized by an inappropriate activation of mesenchymal stem cells in the skeletal muscle tissue, which leads to an extraskeletal bone-containing bone cells, which are derived from several lines. Figure 1 shows the a.p. radiograph of the left hip showing HO formation in the musculus vastus limiting hip flexion and inducing permanent pain. Figure 2 depicts the photograph of the HO after resection and before seperation for different experiments. The histological examination shows the presence of different tissue types, such as mature bones, cartilage and fetal cells. It has been shown that the presence of brown fetal cells reduces the oxygen content and thereby promotes angiogenesis and enchondral ossification, the white fat cells are also present (Fig. 3).

### Flow cytometry

**Fig. 4:**
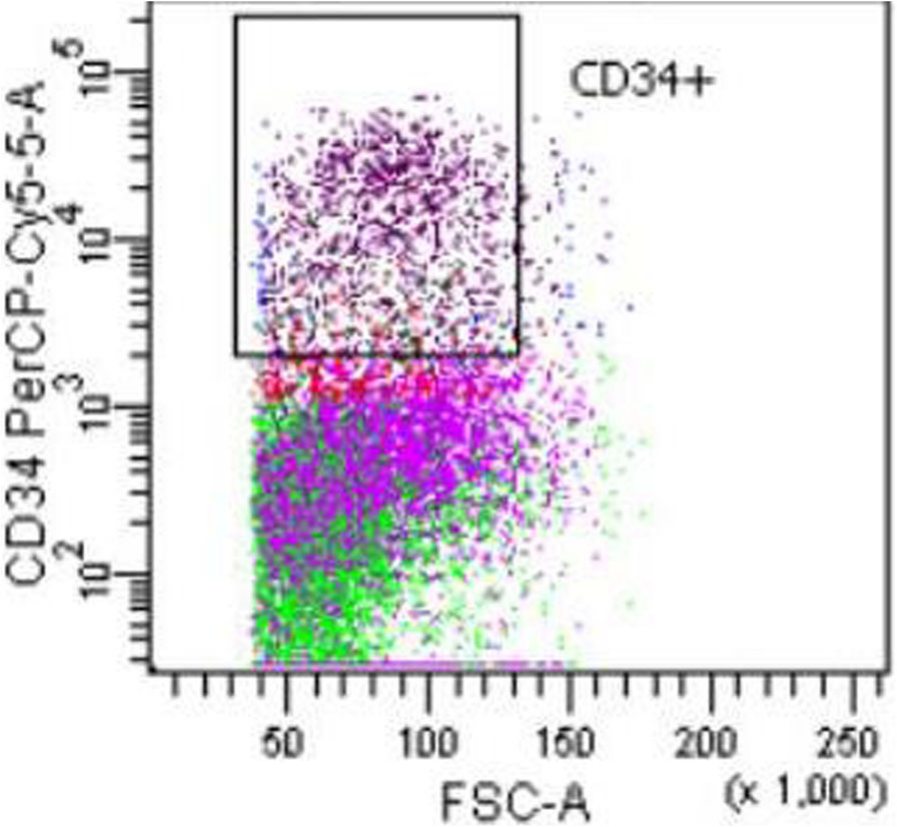
Subpopulations in BMMCs: after excluding dead cells, SSC cells expressing CD34 were gated

**Fig. 5:**
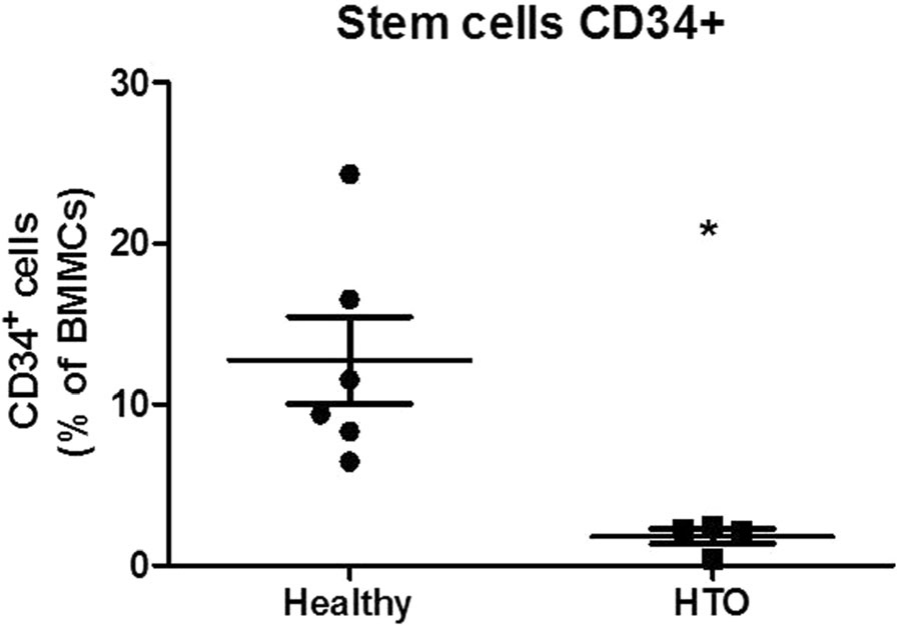
Flow cytometry of the samples (CD 34+ cells)

**Fig. 6:**
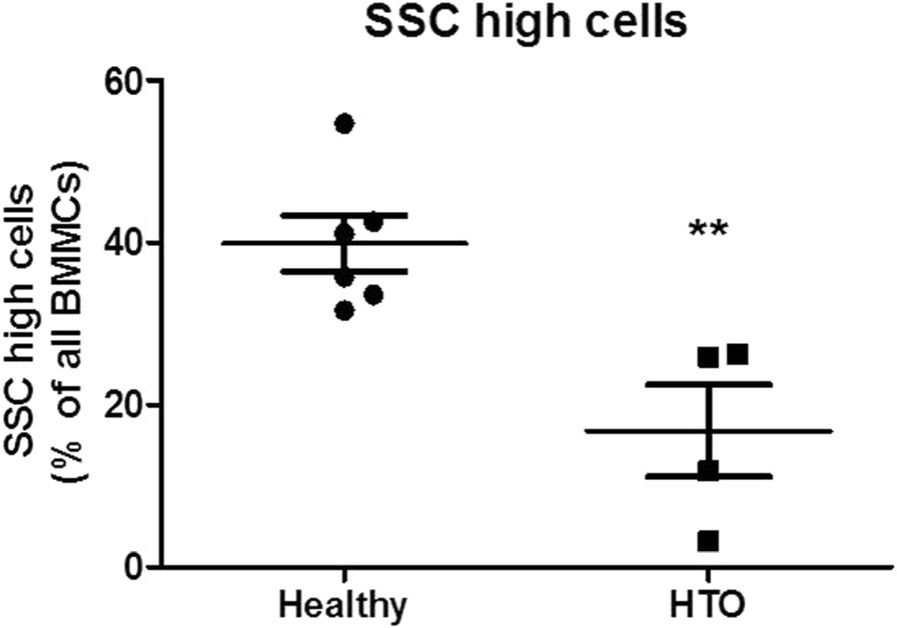
Flow cytometry of the samples (SSC high cells)

Using flow cytometry, the BMMCs of 6 healthy persons can be separated into two populations based on FSC and SSC. The cell population with a low SSC (SSClow) are mainly lymphocytes, monocytes and stem cells, whereas the cells with a higher SSC (SSChigh) are mainly stroma cells and granulocytes progenitor cells. The SSChigh cell population represents 50–70% of all BMMCs but were nearly absent in the patient with heterotopic ossification (Table 1). To further investigate the SSClow population of the BMMCs, we stained with specific markers for monocytes, NK-cells, T cells, B cells and stem cells. The frequency of NK-cells, B cells and T cells were not altered in the patient with HO compared to the healthy controls. However, stromal stem cells and stem cells positive for CD34 were significantly reduced in the HO patients (Figs. 4 and 5). Interestingly we have the same results in the fourth patient comparing normal bone and HTO from the same person. Stromal stem cells (45,4 vs. 25,9) and stem cells positive for CD34 (3,61 vs. 2,09) were reduced in the HO bone, too (Table 1, Fig. 6).

**Table 1:** Flow cytometry analysis of BMMCs

	Healthy		HTO	
	mean	SEM	mean	SEM
T cells	24,72	2.95	24.75	4.08
CD4	46.35	3.16	42.48	9.15
CD8	44.88	3.28	48.73	7.26
B cells	9.357	1.22	13.20	2.43
SSC high	39.9	3.43	16.77**	5.64
CD34+ Stem cells	12.74	2.70	1.77*	0.46
Monocytes	9.57	2.26	4.16	1.58
NK cells	4.87	0.79	5.92	2.95

*p < 0,05; **p < 0,01

### Bone morphometric parameters

Due to the voxel size of 35 μm in the scans of the complete samples, only trabeculae of a thickness larger then 105 μm are considered in the morphometric analysis since at least three voxels are necessary to ascertain that detail detectability is sufficiently high. To investigate the distribution of trabeculae thinner than 105 μm we scanned a cut-out part at a higher resolution at 11 μm voxel size. The values of the extracted microstructural parameters are presented in Table 2.

**Table 2:** Extracted microstructural parameters of the complete sample (35 μm voxel size) and of the cut-out subsample (11 μm voxel size)

	Sample 1	Sample 2	Sample 3	Sample 4
BV/TV (in %)	14,5	39,6	50,9	29,7
TbTh.mean (in μm)	312,1	446,5	352,5	280,5
TbTh.SD (in μm)	192,2	665,5	179,7	135,5
TbSp.mean (in μm)	1634,9	237,9	467,8	1097,3
TbSp.SD (in μm)	1391,5	350,4	312,8	863,8
DA	0,3	0,24	0,34	0,64
TMD (in mg/cm3)	484,3	661,9	635,8	763,5
BMD (in mg/cm3)	109,5	343,6	349,2	283,7

**Fig. 7:**
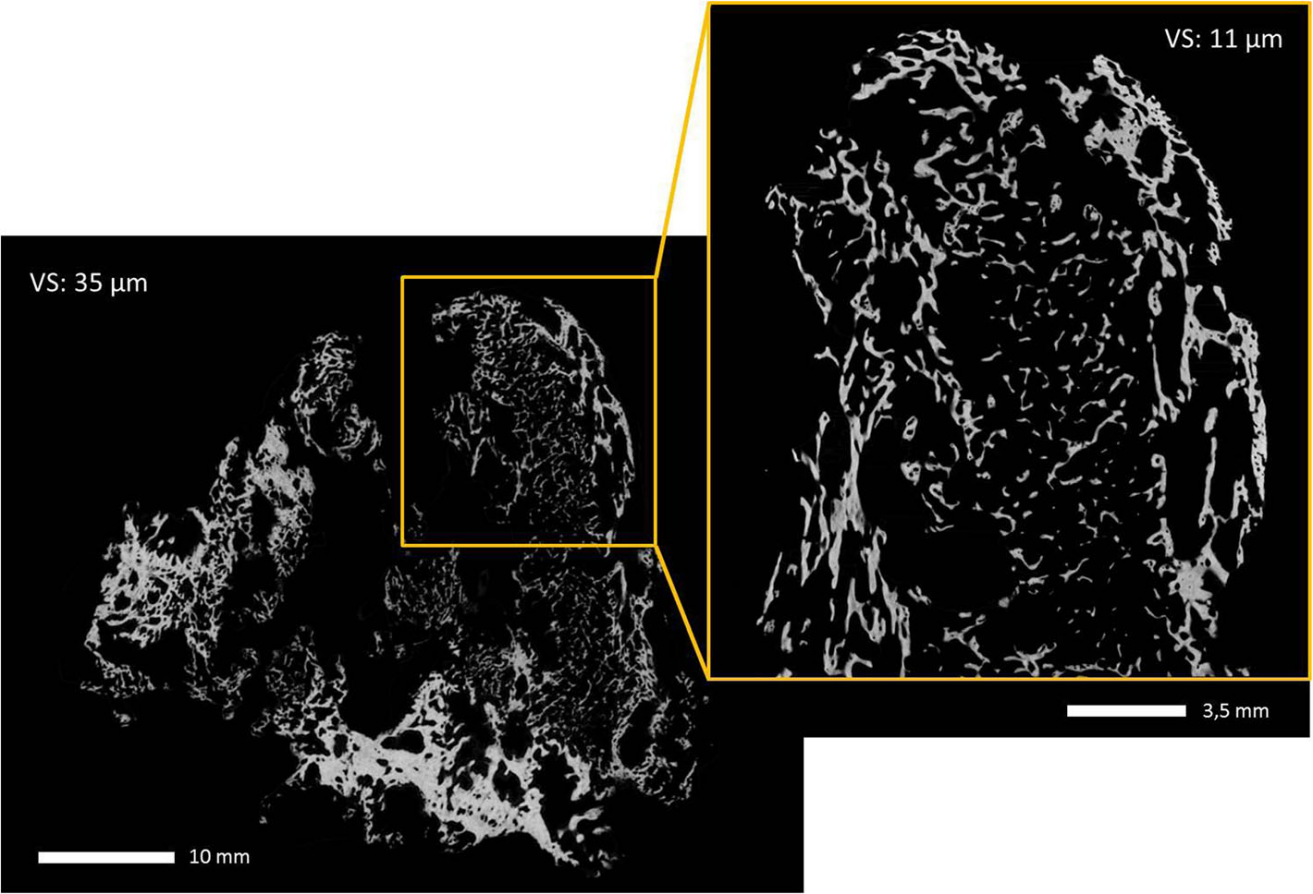
Microcomputed tomography image of one sample

Despite the rather large volume of sample 1 (ca. 24 cm^3^), bone volume fraction (BV/TV) is relative low (14.5%), showing a low content of bone tissue compared to normal 36.46 ± 15.38%), osteoporotic (25.03 ± 6.22%), and metastatic bone (24.29 ± 12.26%) [28]. The high value of mean trabecular separation (TbSp.mean) and its high standard deviation (TbSp.SD) support this finding. Likewise, mean trabecular thickness (TbTh.mean) shows a high standard deviation, indicating a high variation in trabecular thickness from extra skeletal bone to parts of the remaining normal bone adjacent to the resection site. While the central region of the sample shows low values of trabecular thickness, regions at the section site and the outer regions encompassing the central part are characterized by bone structures with a higher trabecular thickness (Fig. 7).

Samples 2–4 show higher BV/TV values between 29,69% and 50,91% (see Tab. 1), exceeding reported values for bone volume fraction [28]. TbSp.mean and TbSp.SD are less pronounced compared to the complete sample 1, showing values between 237.85 μm (Sample 2) and 1097.32 μm (Sample 4) for mean trabecular separation. Depending on the microstructure of the investigated subregion, values for TbSp.mean are either increased (Sample 2) or decreased (Sample 1, 3 and 4). This illustrates the high regional variation in the microstructural composition of the respective sample. The same applies to mean trabecular thickness, Sample 2 and 3 showing higher TbTh.mean values compared to Sample 1. In general, the high standard deviation for TbTh.mean (for overview and detail samples) points to a high variation in the regional distribution of thinner and thicker trabeculae in each sample.

Apart from bone volume fraction, degree of anisotropy (DA) of trabecular bone is another important determinant of biomechanical strength. Using the calculation of DA implemented in CTAn, total isotropy is represented by the value 0 and total anisotropy by 1. In this sample, values between 0.24 and 0.45 point to a rather low low degree of anisotropy, i.e. trabecular alignment along a particular directional axis. However, the low resolution scan of Sample 4 shows a higher DA value. Since, the detail scan of Sample 4 shows a much lower DA value comparable to Samples 1–3, this high value may be explained by very thin trabeculae that are not detected at a lower physical resolution, hence exaggerating the degree of anisotropy. Since thin trabeculae are detected in the detail scan, i.e. those interconnecting larger trabeculae, the total DA is lower. While highly organized bone structures correlate with higher anisotropic values, disordered bone deposition, e.g. of reactive woven bone, is associated with decreased anisotropy [29].

In this study we furthermore quantified the degree of mineralization of bone tissue in the HO sample. Tissue mineral density (TMD) of trabecular bone showed low average values (484.34–763,46 mg HA/cm^3^) compared to average TMD of normal trabecular bone of the femoral neck, greater trochanter, and proximal tibia (approximately 900 mg HA/cm^3^) [30]. Also bone mineral density (BMD) of trabecular bone in conjunction with the surrounding soft tissue showed a lower average value in sample 1 (109,52 mg HA/cm^3^) compared to reported normal bone volumetric BMD for women and men without hip fractures (310 ± 60 mg/cm^3^ and 310 ± 60 mg/cm^3^, respectively) and with hip fractures (250 ± 40 and 260 ± 40 mg/cm^3^, respectively) [31]. Nevertheless, BMD values for Sample 2, 3 and Sample 4 show higher values compared to Sample1.

## Discussion

A neurogenic heterotopic ossification is a serious complication of traumas or disorders of the central nervous system observed in 20% of patients with this condition [2]. The hip and elbow joints affected predominantly are with severe pain, loss of movement and nerve compression syndromes. In addition, complications of the urinary tract system and pressure ulcers may arise in this disease [1–4]. In addition to conservative therapy with NSAIDs or bisphosphonates, surgical resection is indicated, with local recurrence being described.Neurogenic heterotopic ossification is characterized by ectopic bone formation in the soft tissue and muscle tissue around large joints, especially the hip and elbow joints. The severity of the HO depends on the severity of the brain damage. In the initial stage, NHO is difficult to diagnose and can also be interpreted as phlebitis, arthritis or cellulitis in a differential diagnosis, which often leads to a treatment delay. It is then necessary to take care of the hygiene in the case of concomitant diseases and complications as well as, for example, pressure ulcers, urinary tract infections or pneumonia [1–5].

As a rule, surgical resection occurs within the first year after the occurrence of the disease, whereby the indication for the operation is indicated on the one hand by the size of the ossification, on the other also by pain and possible compression of nerves or blood vessels.

Good preoperative planning is important to avoid the potential complications such as infection, fracture, recurrent hemorrhage and nerve injury [2, 20, 21].

The time should be chosen so that the ossification is mature, but not yet so great that the complication probability becomes more frequent. Setting the right time for resection is not always easy, especially given the recurrence probability determined by the severity of brain damage. Likewise, too late a resection is bad for the adjacent joint, since this it is then stiffened and subsequent mobilization is made more difficult. There are different reports in the literature such as a series with 20 hips and another with 29 patients, with an improvement in the scope of movement in both studies [21, 22].

The pathophysiology of the NHO is not fully understood. However, there are 3 causes (traumatic, genetic, neurogenic) that can trigger the formation of the HO by activating stem cells for proliferation and differentiation [23]. In these patients, humoral factors can be altered; the exact relationship between the nervous system and the bone is not fully understood. It has been shown that some factors such as vasoactive peptides, neurotransmitters and the vasoactive substance can be altered [24–27]. There are some limitations in this study, one is the limited number of patients and another the descriptive concept. But our findings should induce other groups to initiate studies on this topic to get more information on the involvement of the immune system in HO.

In our study it is shown that immunological distribution in heterotopic ossification is altered compared to healthy subjects, this might reflect an immunological participation in the development of this entity. The result is tissue formation with a low bone volume fraction. Morphometric parameters additionally show that the disordered bone deposition in HO, e.g. of reactive woven bone, produces bone tissue that is characterized by a decrease in bone strength due to a low degree of mineralization and anisotropy. Further studies are needed to understand the mechanisms which induce HO.

## Conclusions

This work shows altered immunological distribution that is accompanied by a low decrease in bone volume fraction and tissue mineral density in the heterotopic ossification sample compared to normal bone. Compared to healthy subjects, this might reflect an immunological participation in the development of this entity.

## Abbreviations

BM: Bone marrow
BMD: Bone mineral density
BMMC: Bone marrow mononuclear cells
BV/TV: Bone volume/total volume
Con: Connectivity
DA: Degree of anisotropy
HO: Heterotopic ossification
TbSp.mean: Mean trabecular separation
TbSp.SD: Standard deviation of trabecular separation
TbTh.mean: Mean trabecular thickness
TbTh.SD: Standard deviation of trabecular thickness
TMD: Tissue mineral density
